# Surfactant-Free Preparation of Conjugated Polymer Nanoparticles in Aqueous Dispersions Using Sulfate Functionalized Fluorene Monomers

**DOI:** 10.1021/jacs.4c08985

**Published:** 2024-09-19

**Authors:** Marcin Gwiazda, Benjamin J. Lidster, Charlotte Waters, Jaruphat Wongpanich, Michael L. Turner

**Affiliations:** Department of Chemistry, University of Manchester, Oxford Road, Manchester M13 9PL, U.K.

## Abstract

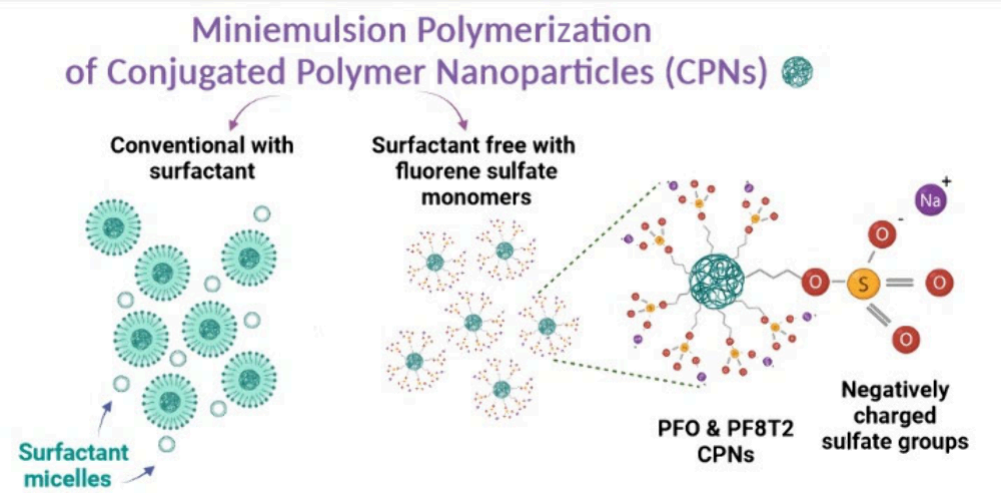

Conjugated polymer nanoparticles (CPNs) can be synthesized by a Suzuki–Miyaura cross-coupling miniemulsion polymerization to give stable dispersions with a high concentration of uniform nanoparticles. However, large amounts of added surfactants are required to stabilize the miniemulsion and prevent the aggregation of the nanoparticles. Removal of the excess surfactant is challenging, and residual surfactant in thin films deposited from these dispersions can reduce the performance of optoelectronic devices. We report a novel approach to prepare stable dispersions with no added surfactant using a fluorene monomer, 2,7-dibromo-9,9-bis(undecanesulfate)-9*H*-fluorene, with alkyl side chains terminated by negatively charged sulfate groups. This functionality mimics the structure of one of the most commonly used surfactants, sodium dodecyl sulfate (SDS). This charged monomer effectively stabilizes the miniemulsion through electrostatic repulsion without the use of any additional surfactant in molar ratios ranging from 2.0 to 20.0 mol % of total monomer content for the preparation of poly(9,9-dioctylfluorene) (PFO) and poly(9,9-dioctylfluorene-*alt*-bithiophene) (PF8T2). Incorporation of 5.0 mol % of the amphiphilic monomer gave stable dispersions with a surface potential below −40 mV and, and polymers with molar mass (*M*_*n*_) above 10 kg mol^–1^. This method should be generally applicable to the preparation of dispersions of polyfluorenes for application in organic electronic and optoelectronic devices without the requirement for time-consuming processes to remove residual surfactant.

## Introduction

Conjugated polymers exhibit many desirable electronic and optoelectronic properties, such as the efficient transport of charge carriers,^1,2^ a tunable band gap, intense optical absorption, high photostability and high photoluminescence quantum yields.^3−6^ In addition these are coupled with the attractive physical properties of macromolecules such as mechanical durability^7^ and flexibility^8,9^ This combination leads to a wide spectrum of possible applications in devices such as organic photovoltaics (OPVs), organic field-effect transistors (OFETs), organic light emitting device (OLEDs), and in biological imaging or biosensing.^10−13^ The hydrophobic nature of conjugated polymers requires the use of undesirable and even toxic organic solvents for processing of these materials, such as chlorinated aromatics.^14^ It is essential to improve the environmental profile for processing of these materials into devices, and to enable the use in bioimaging applications it is necessary to disperse conjugated polymers in a more biocompatible medium such as water.^14^ There are several different strategies for dispersing conjugated polymers in water such as nanoprecipitation,^15,16^ dispersion or emulsification with a surfactant,^17−19^ crystallization-driven self-assembly (CDSA)^20,21^ and the use of microfluidic devices.^22,23^ Using these approaches it is possible to control the size, morphology, and concentration of the conjugated polymer nanoparticles (CPNs).

Nanoprecipitation involves dissolving a conjugated polymer in an organic solvent that is miscible with the aqueous phase.^24,25^ This polymer solution is directly dosed into a large excess of water, and CPNs are formed with a small size (generally <30 nm).^26−28^ This approach generally leads to a low concentration of CPNs and has poor scalability, and in general the dispersions have limited long-term stability.^29−31^ These CPN dispersions can be stabilized by adding a surfactant such as SDS or polystyrene-*co*-maleic anhydride (PSMA).^32,33^ For example Kosco et al. prepared PSMA stabilized dispersions of PF8BT by nanoprecipitation to give CPNs in the range of 50 nm that can be utilized for photocatalytic water splitting.^16^ The presence of excess surfactant in these dispersions is generally detrimental to the processing of these materials and also the properties of thin films deposited from these semiconductors.^34,35^ The excess of ionic surfactants such as SDS can be removed through a time-consuming process of dialysis or centrifugal filtration.^34^ This technique was used to prepare CPN dispersions from a solution of PTQ10 and PC_61_BM in chloroform stabilized using SDS surfactant.^36^ By matching the free surface energy between donor (PTQ10) and acceptor (PC_61_BM) materials, it is possible to prepare homogeneous distribution in composite CPNs suitable for deposition of nanostructured thin films to act as bulk heterojunction organic photovoltaics. These devices show power conversion efficiencies approaching 10%.^36^ Nonionic poloxamer surfactants can be removed by centrifugation and filtration at low temperatures.^35^ Thin films of PBQ-QF:ITIC deposited from these dispersions showed improved power conversion efficiency (PCE) in the photovoltaic device when compared to the material processed without the surfactant-stripping process (7.5% vs 5.2%). This technique was also employed to prepare donor (IDTBT) and acceptor (oIDTBR) composite nanoparticles with an average size of 70 nm that showed effective photocatalytic water splitting.^37^

An alternative approach is to prepare the polymer directly in a water immiscible dispersed phase by miniemulsion polymerization. In this approach the organic phase is added directly to the aqueous phase with a high concentration of surfactant.^38^ Sonication of the mixture forms nanoreactors in which the polymerization takes place that are stabilized by the surfactant, and evaporation of the solvent leaves a dispersion of conjugated polymer nanoparticles in the aqueous phase.^38^ Advantages of the miniemulsion process include the preparation of uniform nanoparticles ranging in size from 20 to 500 nm^21^ in a one-pot process with a relatively high concentration of nanoparticles (>1 mg cm^–3^) and in some cases control of the nanoparticle morphology.^18^ Miniemulsion polymerization of CPNs has been achieved by addition of various surfactants, including sodium dodecyl sulfate (SDS)^39^ and tetradecyltrimethylammonium bromide (TTAB)^40^ or nonionic surfactants such as Triton X.^18^ For these dispersions to be useful for the deposition of conjugated polymer thin films the excess of surfactant has to be removed by dialysis.^41,19^ p-Type and n-type OFETs have been fabricated using PIDTBT and PDPPTBT films deposited from CPN dispersions, prepared by miniemulsion polymerization. The performance of these materials in device (μ = 0.18 and 0.36 cm^2^ V^–1^ s^–1^, respectively) is essentially identical to those of materials processed by conventional methods but with a reduction of nearly 2 orders of magnitude in the amount of organic solvent employed.^34^ The amount of solvent added in the miniemulsion polymerization can be further reduced by choice of surfactant system. Sanzone et al.^42^ synthesized CPNs based on PF8T2 and PF8BT in ambient conditions, using the nonionic Kolliphor EL (K-EL) as a surfactant, with a minimal addition of toluene solvent. Similar approaches were implemented by Mattiello et al.^43^ for Suzuki–Miyaura cross-coupling reactions and Calascibetta et al.^44^ for the synthesis of thiophene-containing π-conjugated building blocks. Recently Beverina’s group^45^ has shown that a mixture of amphiphiles, Tween 80 and l-α-lecithin, can be used to prepare PF8T2 dispersions in the absence of added solvent. These dispersions can be used to spin coat the active layer in an OFET, leading to devices with a saturation hole mobility of 1 × 10^–3^ cm^2^ V^–1^ s^–1^.^46^ Surfactant-free approaches to prepare CPN dispersions have been reported using rod–coil block copolymers, processed into the aqueous phase due to the incorporation of the hydrophilic 4-vinylpyridine (4VP) segments.^41^ CPN dispersions were prepared by nanoprecipitation where the core of the nanoparticle was composed of the conjugated polymer, PCPDTBT, that is surrounded by a shell of 4VP. This approach gives stable colloidal dispersion of NPs, but it requires the preparation of the conjugated block copolymer with 2, 5, or 15 mol % of the 4VP segment and this will have a considerable impact on the physical and optoelectronic properties of the material. An alternative approach was reported by Marcial-Hernandez et al.^47^ by substitution of oligoether side chains onto P3HT to form CPNs dispersions. Incorporation of 10–20 mol % of PEO chains gave dispersions that were stable for more than one month. These approaches to high solids loading, surfactant-free dispersions require significant synthetic polymer chemistry efforts, and it is highly desirable to develop improved approaches to stable dispersion of CPNs with a narrow size distribution, high molecular weight, and without significant modification of the main polymer backbone chain, to maintain the desirable optoelectronic properties of the polymers when deposited in thin films.

Another effective way to stabilize aqueous dispersions of CPNs was developed by Creamer et al.;^48^ this involved the nucleophilic substitution of PEG chains for fluoride on the backbone of poly(9,9′-dioctylfluorene-5-fluoro-2,1,3-benzothiadiazole). The resulting polymers could be redispersed in THF solution and injected into an excess of water to form CPNs by nanoprecipitation. Longer PEG chains reduced the tendency of the nanoparticles to agglomerate, leading to high colloidal stability and nanoparticles below 30 nm in diameter.

In this study, we report the synthesis of amphiphilic fluorene monomers terminated with negatively charged sulfate groups at the end of the alkyl side chain. These monomers enable surfactant-free miniemulsion polymerizations to prepare poly(9,9′-dioctylfluorene) (PFO) and poly(9,9-dioctylfluorene-*alt*-bithiophene) (PF8T2) using a minimal amount of the sulfate fluorene monomer. The molar ratio of the added sulfate fluorene monomer to achieve a homogeneous dispersion of the nanoparticles was examined, and the dispersions were shown to be stable for many months in water. This amphiphilic monomer can replace the use of added SDS surfactant but does not significantly change the chemical composition and final properties of the polyfluorenes. As a result, this feature could have potential applications in future organic electronic devices based on CPNs without surfactant.

## Results and Discussion

**Scheme 1:**
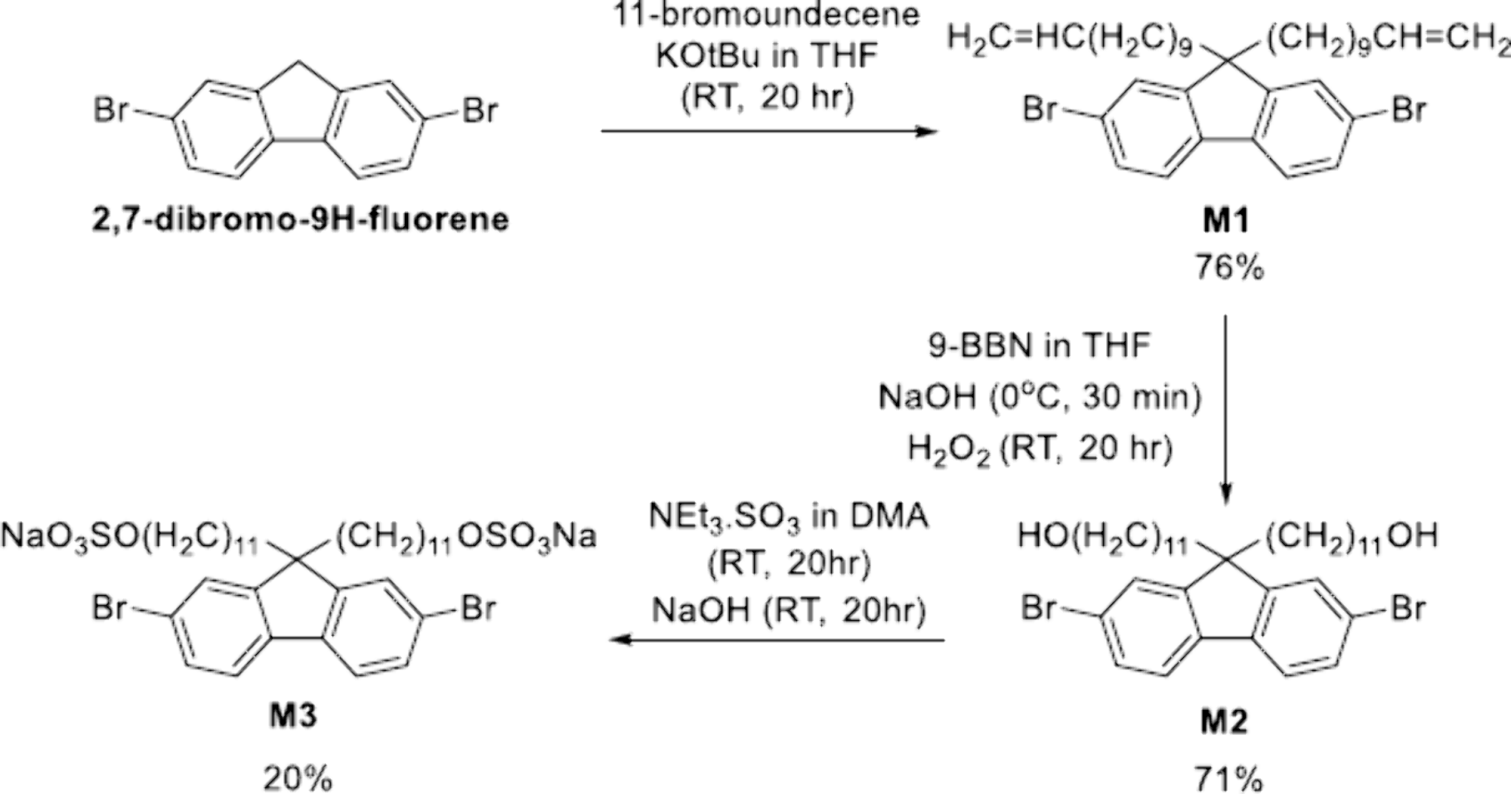
Synthetic Route To Prepare Monomer **M3**

The amphiphilic fluorene monomer (**M3**) was synthesized in three steps from 2,7-dibromofluorene by alkylation of the 9-position, hydroboration and sulfonation, as shown in Schemes 1 and S1–S3.

**Scheme 2:**
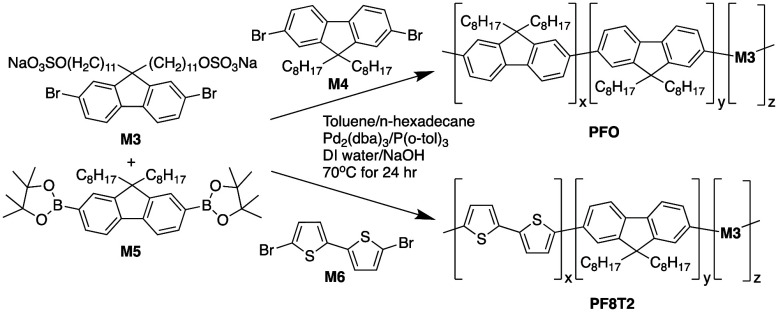
Synthesis of Aqueous Dispersions of **PFO** and **PF8T2** Nanoparticles Using Alkyl-Sulfate Functionalized Fluorene Monomer **M3**

Monomer **M3** is substituted with two sulfate groups per fluorene ring and is poorly soluble in toluene but soluble in polar solvents such as water and dimethyl sulfoxide. It was found to effectively stabilize a toluene/water emulsion, generated by sonication, to enable palladium catalyzed Suzuki–Miyaura cross-coupling polymerization with an equimolar ratio of bromine to boronate ester groups from **M3**, **M4** or **M6** and **M5**, respectively (Scheme 2). Stable dispersions of **PFO** (using **M4**) and **PF8T2** (using **M6**) nanoparticles could be prepared in the presence of added hexadecane by reaction at 70 °C for 24 h followed by removal of the residual toluene by nitrogen purging. Incorporation of **M3** in the range of 2 to 20 mol % of total added monomer generated a homogeneous dispersion of conjugated polymer nanoparticles with a concentration of CPNs of approximately 5 mg cm^−3^. Dialysis was employed to remove the byproducts of the Suzuki–Miyaura polymerization such as boronic acid and sodium bromide. It is not required to remove an excess of added surfactant but simply to exchange the water with clean deionized water.

**Figure 1:**
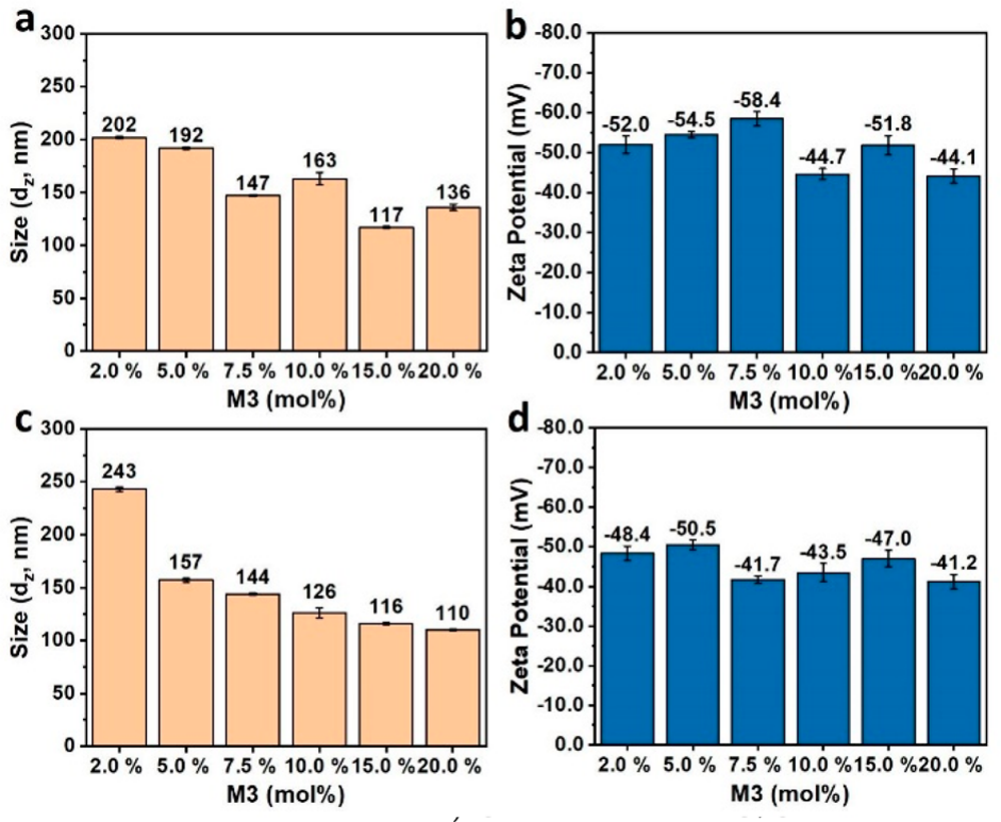
Influence of **M3** molar composition (2.0, 5.0, 7.5, 10.0, 15.0, and 20.0 mol %) on z-average nanoparticle diameter (*d*_*z*_), and surface zeta-potential (mV) for **PFO** (a and b) and **PF8T2** (c and d) dispersions.

The average hydrodynamic diameter of the nanoparticles and the surface zeta-potential are summarized in Figure 1. Higher incorporations of **M3** led to a smaller size of the nanoparticles as the average diameter of the **PFO** CPNs decreased from 202 nm for 5 mol % of **M3** to 136 nm for 20 mol %. The same trend was observed for the **PF8T2** CPNs where the size of the nanoparticles was halved on increasing **M3** from 2 to 20 mol %. The CPN dispersions show an isotropic structure and uniform dimensions as *PDI*_*DLS*_ are low, ranging from 0.13 to 0.26.

The zeta potential of the dispersions prepared using **M3** ranged from −41 to −58 mV (Figure 1); this is consistent with a stable dispersion predicted by Derjaguin–Landau–Verwey–Overbeek (DLVO) theory^49^ that requires a surface potential of the dispersed phase below −40 mV for colloidal stability using an anionic surfactant.^50−52^

DLS analysis of all samples after one month (Figure S4) showed essentially identical sizes and zeta-potentials to the initial measurements, confirming that addition of the sulfate fluorene monomers during miniemulsion polymerization for **PFO** and **PF8T2** gives highly stable CPNs dispersions.

Samples of the **PFO** and **PF8T2** polymers were isolated from the dispersions by precipitation using methanol (S2.4). The molecular masses of the polymers were determined by SEC and are summarized in Table 1. The number-average molar mass (*M*_*n*_) decreased with incorporation of the sulfate-containing monomer, **M3**. The dispersities (*Đ)* of the polymers were above 2. The molar masses are higher than those in previously reported studies on the preparation of homobifunctional (*M*_*n*_ = 15 kg mol^–1^) and heterobifunctional (*M*_*n*_ = 9 kg mol^–1^) Suzuki–Miyaura cross-coupling polymerization of polyfluorenes in a miniemulsion stabilized by added surfactant.^17,18,53^ Incorporation of more than 10 mol % of sulfate monomers led to a significant drop in the observed molecular mass (*M*_*n*_) below 10 kg mol^–1^, presumably due to a stoichiometric imbalance at the small scale of the reactions and the partial solubility of monomer **M3** in water, with added **M3** remaining in the aqueous phase rather than completely partitioning to the organic phase. It should also be noted that higher molecular weight polymers with high loadings of the sulfate side groups (15 and 20 mol %) are poorly soluble in the THF solvent used for the SEC measurements, hence leading to lower than expected molecular weights.

**Table 1:** Summary of Molecular Weight with Polydispersity, Maximum of Optical Absorbance, and Percentage of β-Phase for the Synthesized **PFO** and **PF8T2** CPNs Samples with Various Ratios of Sulfate Fluorene Monomers (2.0, 5.0, 7.5, 10.0, 15.0, and 20.0 mol %)

CPNs	**M3** (mol %)	*M*_*n*_ (g/mol)	*Đ*	*λ*_*max*_ (nm)	β-phase (%)
**PFO**	2.0	14,450	3.67	403	20.0
	5.0	20,460	3.31	401	15.2
	7.5	9,870	2.61	391	7.2
	10.0	12,050	2.87	395	7.7
	15.0	8,750	3.08	387	2.6
	20.0	5,010	3.04	386	3.3
					
**PF8T2**	2.0	14,970	3.27	496	–
	5.0	11,700	2.73	457	–
	7.5	11,290	2.33	456	–
	10.0	12,320	3.59	448	–
	15.0	4,930	3.43	443	–
	20.0	2,740	1.68	440	–

**Figure 2:**
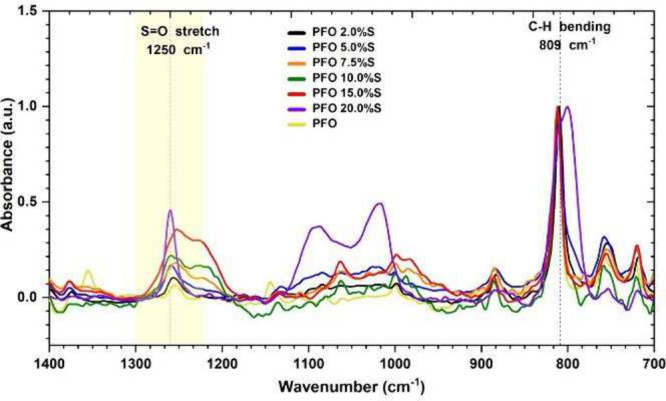
ATR-FTIR spectra were recorded for **PFO**.

The incorporation of the charged monomer **M3** into the polymer backbone was examined by ATR-FTIR spectroscopy, and the vibrational spectra of the solid polymers, **PFO** and **PF8T2**, are presented in Figure 2 and Figure S5.

Peaks at 1,250, 1,073 and 958 cm^–1^ were assigned to S=O stretching of the sulfate groups derived from **M3**.^54^ To quantify the incorporation of **M3** into the polymer backbone, the intensity of the absorption at 1,250 cm^–1^ was normalized to the peak at 809 cm^–1^ that is associated with the out-of-plane, aromatic C–H bending (Figure S6).^55^

The normalized absorbance of the sulfate peak at 1250 cm^–1^ was directly proportional to the ratio of added **M3** monomer for both **PFO** and **PF8T2**, confirming that **M3** was incorporated into the main chain of both polymers in direct proportion to the ratio of added monomer during the polymerization process. In the ^1^H NMR spectra of these polymers a triplet peak at δ 3.70 ppm was assigned to the hydrogens associated with the methylene group attached to the sulfate^56^ (Figures S7–S9).

The peak was detected for all of the polymer samples, but the poor solubility limited the possible analysis as it was difficult to find a common solvent in which all of the polymers, 2–20 mol % of **M3**, were soluble.

**Figure 3:**
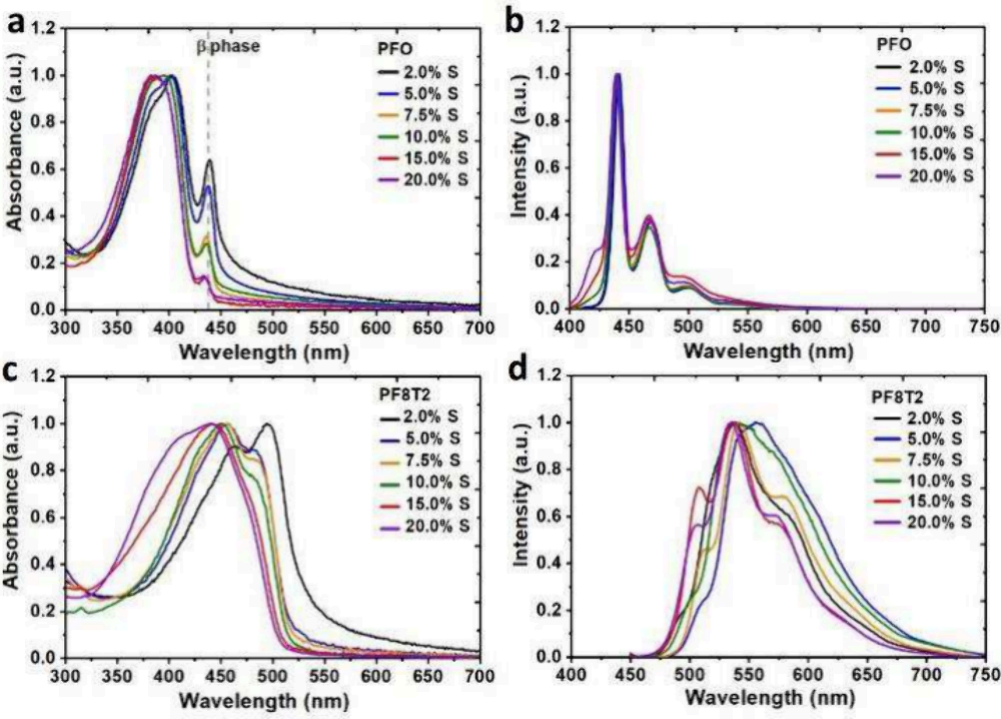
Absorption (UV/vis) and photoluminescence (PL) spectra for **PFO** (a and b) and **PF8T2** (c and d) CPNs dispersed in an aqueous solution.

The optical absorption and emission spectra of the **PFO** and **PF8T2** CPN dispersions are presented in Figure 3, and the maximum absorbance values are summarized in Table 1. The results obtained are consistent with previously reported studies on analogous polyfluorene CPN dispersions.^17,18,57^ Two superimposed peaks are observed in the UV/vis absorption spectra of the **PFO** dispersions, the disordered amorphous phase (*λ*_*max*_) in the range from 386 to 403 nm and the ordered β-phase at *ca*. 430 nm.^58^ The planar conformation of the β-phase of **PFO** reduces the optical band gap, leading to a red-shifted emission band, an improvement in the hole/electron mobility and an increase in the extinction coefficient.^59,60^ The blue shift of the maximum absorbance from 403 nm for **PFO** CPNs with 2 mol % **M3** to 386 nm for 20 mol % **M3** is consistent with a higher proportion of amorphous **PFO** in nanoparticles with higher loadings of **M3**. The absorption spectra of the **PFO** CPNs were fitted by a bigaussian function, which allows for the deconvolution^61^ of the composite peak into the individual contributions from the *λ*_*max*_ and β-phase absorptions (Figure S10). The proportion of β-phase component decreased linearly with composition until it reached a plateau at approximately 15% of **M3** (Figure S11, Table S3).^18^ In the case of the **PF8T2** CPNs, the maximum absorption ranges from 440 to 496 nm, with a blue shift on increasing the ratio of the added **M3** and a greater proportion of glassy polymer in the nanoparticles. Dispersions with 2 and 5 mol % of added **M3** showed a characteristic peak at approximately 500 nm for a more ordered phase of **PF8T2** that decreases with higher incorporation of **M3**.^18^

**Figure 4:**
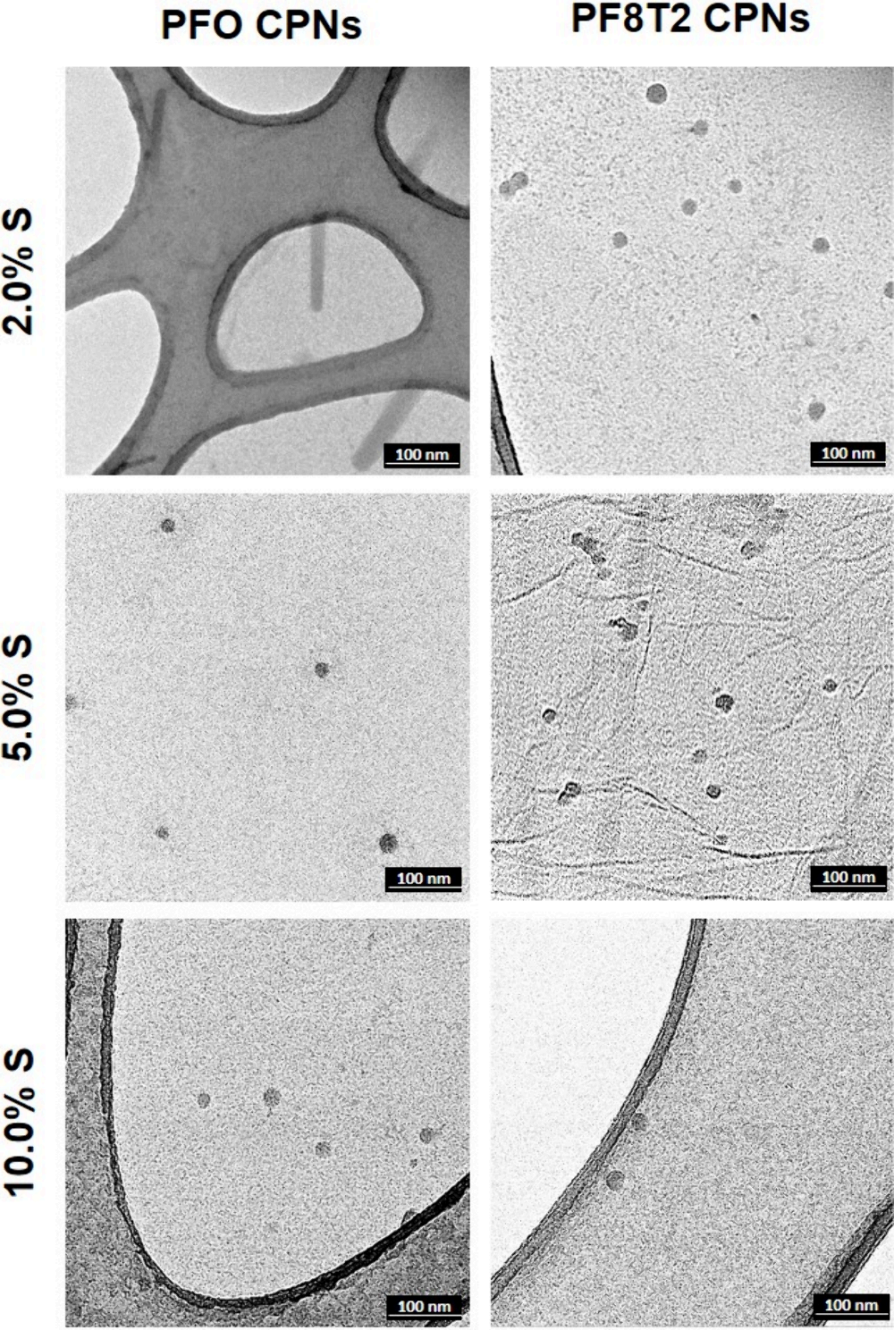
TEM images of the **PFO** and **PF8T2** with 2.0, 5.0, and 10.0 mol % of the addition of the sulfate fluorene monomers acquired under 92,000× magnifications in bright field.

The morphologies of the **PFO** and **PF8T2** nanoparticles, synthesized by addition of **M3** (2.0, 5.0, 10.0, and 20.0 mol %), were examined by TEM, and the results are shown in Figure 4 and Figure S12. All nanoparticles are spherical except the **PFO** dispersions made with 2.0 mol % **M3**, and these nanoparticles were anisotropic rods of around 100 nm in length and 20 nm width with an aspect ratio of 5:1. Similar rod-shaped nanoparticles have been observed for **PFO** dispersions synthesized using a high concentration of nonionic surfactants such as Triton X102.^18,62^

For the spherical nanoparticles of **PFO** and **PF8T2** the measured diameter (*d*_*n, TEM*_) for higher loadings of **M3** is smaller than those with lower loadings (Table 2), and this is consistent with the trend in diameter measured by DLS (*d*_*n, DLS*_). But there is a large difference in the absolute values of the diameter measured by DLS and TEM due to high vacuum conditions of the TEM experiment. The size distributions of the CPNs are compared in Figure S13 and Figure S14, and a histogram of the distribution of the rod shaped **PFO** nanoparticles is shown in Figure S15.

**Table 2:** Summary of the Average Diameter of **PFO** and **PF8T2** CPNs with the Addition of **M3** Recorded by DLS (Number Distributions, *d*_*n,DLS*_) with Polydispersity *PDI*_*DLS*_ and Determined from TEM Images (*d*_*n, TEM*_)^a^

CPNs	**M3** (mol %)	*d*_*n, DLS*_ (nm)	*PDI*_*DLS*_	*d*_*n, TEM*_ (nm)
PFO	2.0	134 ± 11	0.14 ± 0.02	21 ± 6 (d)
				113 ± 40 (l)
	5.0	165 ± 8	0.17 ± 0.03	27 ± 11
	10.0	77 ± 11	0.26 ± 0.06	28 ± 9
	20.0	76 ± 5	0.17 ± 0.02	10 ± 7
				
PF8T2	2.0	133 ± 16	0.22 ± 0.01	32 ± 8
	5.0	113 ± 8	0.13 ± 0.02	25 ± 7
	10.0	28 ± 3	0.26 ± 0.01	32 ± 7
	20.0	63 ± 14	0.16 ± 0.02	19 ± 6

a The diameter (*d*) and length (*l*) of the rod shaped nanoparticles were measured for **PFO** 2.0% S CPNs.

## Conclusion

Stable, aqueous nanoparticle dispersions of the conjugated polymers, **PFO** and **PF8T2**, have been prepared using a surfactant-free miniemulsion polymerization. This was achieved using an alkyl-sulfate functionalized fluorene monomer, **M3**, prepared from readily available 2,7-dibromofluorene. The amphiphilic monomer is similar in structure to the most commonly applied anionic surfactant, sodium dodecyl sulfate (SDS), but is incorporated into the conjugated polymer backbone at loadings ranging from 2.0 to 20.0 mol %. Even the lowest loading of added **M3** gave stable dispersions with ζ-potentials less than −40 mV. The size of the nanoparticles decreased with a higher proportion of added **M3**, and dispersions synthesized with 5 or 10 mol % of **M3** gave spherical 150 nm nanoparticles with polymer molecular weights >10 kDa. The incorporation of the alkyl-sulfate fluorene monomer was confirmed by both ^1^H NMR and ATR-FTIR spectroscopy. This surfactant-free approach was used to prepare stable dispersions of polyfluorene homopolymers (**PFO**) and copolymers with bithiophene (**PF8T2**). The use of these dispersions to deposit thin films for use in optoelectronic devices is currently under investigation.

## Supporting Information Available

Experimental section with used materials (S1.1), method of the synthesis of the **M1**, **M2** and **M3** monomers (S1.2–4) and polymers (S.1.5), ^1^H and ^13^C NMR spectra for **M1**, **M2** and **M3** compounds (Figures S1–S3), description of the measurement setup (S.2.1–6) for the material characterisations (DLS, UV/vis, SEC, FTIR, TEM), sample preparation with different addition of **M3** monomers for **PFO** (Table S1) and **PF8T2** CPNs (Table S2), stability of **PFO** and **PF8T2** CPNs (Figure S4), FTIR spectra for **PF8T2** (Figure S5), normalized absorbance for S=O stretch from FTIR spectra for **PFO** and **PF8T2** (Figure S6), ^1^H NMR spectra for all **PFO** and **PF8T2** samples (Figures S7–S9), deconvoluted UV/vis absorption spectra for all **PFO** CPNs samples (Figure S10), β-phase contribution for all **PFO** samples (Figure S11), integration area of peaks corresponding to α– and β-phases for all **PFO** CPNs samples (Table S3), TEM images of **PFO** and **PF8T2** CPNs with 20.0 mol % S (Figure S12), distributions of CPNs size determined form DLS and TEM for all PFO (Figure S13) and PF8T2 (Figure S14) CPNs, histogram of the length of rod CPNs for PFO with 2.0 mol % S (Figure S15). (PDF)
