# Supplementary material for: Surfactant-Free Preparation of Conjugated Polymer Nanoparticles in Aqueous Dispersions Using Sulfate Functionalized Fluorene Monomers
Source: J Am Chem Soc. 2024 Sep 19;146(39):27040–6. doi: 10.1021/jacs.4c08985 (PMC11450809; doi:10.1021/jacs.4c08985)
Supplement: Supplementary file 1 — ja4c08985_si_001.pdf [file ja4c08985_si_001.pdf]

Supporting Information

Surfactant-Free Preparation of Conjugated Polymer Nanoparticles in Aqueous Dispersions Using Sulfate Functionalized Fluorene Monomers

Marcin Gwiazda, Benjamin Lidster, Charlotte Waters, Jaruphat Wongpanich, Michael L. Turner*

Department of Chemistry, University of Manchester, Oxford Road, Manchester M13 9PL, UK

S.1 Experimental

S.1.1 Materials

9,9-Dioctyl-2,7-dibromofluorene, 9-borabicyclo[3.3.1]nonane (9-BBN), N,N dimethylacetamide (DMA), hexadecane, and sodium dodecyl sulfate (SDS) were supplied from Sigma-Aldrich. 11-Bromo-1-undecene, potassium *tert*-butoxide, sulfur trioxide trimethylamine, and tris(dibenzylideneacetone)dipalladium(0) [$Pd_2(dba)_3$] were purchased from Fluorochem. 5,5′-Dibromo-2,2′-bithiophene, tri(o-tolyl)phosphine ($P(o\text{-}tol)_3$) were obtained from Acros Organics, 2,7-dibromofluorene from Apollo Scientific, and 9,9-dioctylfluorene-2,7-bis(boronic acid pinacol) ester was from Alfa Aesar. All received commercially available chemical reagents were used without any additional purification.

S.1.2 Synthesis of 9,9-Di(undec-10-en-1-yl)-2,7-dibromofluorene (M1)

11-Bromo-1-undecene (16.3 mL, 17.3 g, 74.1 mmol) was added into a solution of 2,7-dibromo-9H-fluorene (10.0 g, 30.9 mmol) and potassium *tert*-butoxide (13.9 g, 123.6 mmol) in dry tetrahydrofuran (THF) (150 mL) at 0°C. The reaction mixture was stirred at room temperature for 20 hours (**Scheme 1**) and then diluted with 500 mL of deionized water, neutralised using 1M HCl, and extracted using ethyl acetate. The collected organic phase was dried over magnesium sulfate, and the residual solvents were evaporated *in vacuo*. The crude product was purified by recrystallisation from isopropanol (IPA), and isolated by filtration through a Buchner funnel to yield a white powder product (14.71 g, 23.6 mmol, 76%). The NMR spectra are shown in **Figure S1**. ^{1}H NMR (400 MHz, $CDCl_3$) δ 7.53-7.44 (m, 6H, H aromatic), 5.84-5.74 (m, 2H, H alkene), 5.00-4.90 (m, 4H, H alkene), 2.03-1.89 (m, 8H), 1.54-1.05 (m, 24H), 0.57 (s, 4H).^{13}C NMR (100 MHz, $CDCl_3$) δ 152.68 (aromatic), 139.40 (aromatic), 139.21 (alkene), 130.29 (aromatic), 126.31 (aromatic), 121.61 (aromatic), 121.27 (aromatic), 114.21 (alkene), 55.82 (quaternary), 40.29, 33.93, 29.98, 29.59, 29.50, 29.31, 29.21, 29.04, 23.76; HRMS (EI) Calcd. for $C_{35}H_{48}Br_2$ [M^+] 626.2117, found 626.2130.

S.1.3 Synthesis of 9,9-Di(undecane-1-ol)-2,7-dibromofluorene (M2)

9,9-Di(undec-10-en-1-yl)-2,7-dibromofluorene (2.00 g, 3.18 mmol, **Scheme 1**) was dissolved in anhydrous THF (5 mL) and 9-borabicyclo[3.3.1]nonane (9-BBN) was added as a 0.5M solution in THF (15.3 mL, 7.64 mmol), followed by heating at reflux for 5 hours. The reaction mixture was cooled to room temperature, and 10 mL of 3M NaOH aqueous solution was slowly added at 0°C, followed by 8 mL of H_2O_2 (30 wt.%). The solution was vigorously

stirred for 24 hours and then neutralised using 1M solution of HCl. The solution was extracted three times by THF (3 × 100 mL) and brine (3 × 100 mL), then the collected organic phase was dried over magnesium sulfate. The organic solvent was evaporated in *vacuo* to leave a yellow oil that was purified by silica column chromatography using an eluent containing chloroform and methanol (98:2) ratio to yield a colourless oil product (1.50 g, 2.26 mmol, 71%). The NMR spectra are shown in the Supplementary Information in **Figure S2**. ^{1}H NMR (400 MHz, $CDCl_3$) δ 7.53-7.43 (m, 6H, H aromatic), 3.64-3.61 (t, J = 6.7 Hz, 4H), 1.92-1.88 (m, 4H), 1.58-1.51 (m, 4H), 1.33-1.04 (m, 28H), 0.58 (s, 4H).^{13}C NMR (100 MHz, $CDCl_3$) δ 152.55 (aromatic), 139.07 (aromatic), 130.15 (aromatic), 126.17 (aromatic), 121.46 (aromatic), 121.14 (aromatic), 63.12 (C-OH), 55.68 (quaternary), 40.14, 32.79, 29.84, 29.51, 29.47, 29.44, 29.37, 29.17, 25.70, 23.61; HRMS (ESI) Calcd. for $C_{35}H_{52}Br_2O_2Na$ [M^+] 685.2226, found 685.2209.

S.1.4 Synthesis of 9,9-Di(undecanesulfate)-2,7-dibromofluorene (M3)

9,9-Di(udecane-1-ol)-2,7-dibromofluorene (1.00 g, 1.59 mmol) and sulfur trioxide trimethylamine (0.65 g, 3.82 mmol) were dissolved in dry *N*,*N* dimethylacetamide (20 mL) and stirred for 20 hours at room temperature. An excess of NaOH (3M, 20 mL) was added and the solution stirred for an additional 20 hours at room temperature (**Scheme 1)**. 9,9-Di(undecanesulfate)-2,7-dibromofluorene (**M3**) precipitated and was collected by filtration followed by washing with water (2 × 50 mL), ethanol (2 × 50 mL), and chloroform (2 × 50 mL) to give white crystals (276 mg, 0.318 mmol, 20%). The NMR spectra are presented in **Figure S3**. ^{1}H NMR (400 MHz, DMSO-d^6) δ 7.79-7.51 (m, 6H, H aromatic), 3.65 (t, 4H, CH_2OSO_3), 2.02-1.98 (m, 4H), 1.47-1.41 (m, 4H), 1.31-0.99 (m, 28H), 0.42 (s, 4H).^{13}C NMR (100 MHz, DMSO-d^6) δ 152.91 (aromatic), 139.31 (aromatic), 130.51 (aromatic), 126.51 (aromatic), 122.49 (aromatic), 121.42 (aromatic), 65.95 (C-OSO_3Na), 56.03 (quaternary), 39.14, 29.54-23.61; HRMS (ESI) Calcd. for $C_{35}H_{50}Br_2O_8S_2$ [M^+] 410.0662 (z=2), found 410.0654; FT-IR: 2,930 cm^{-1} (C-H stretch), 1,235 cm^{-1} (S=O stretch).

S.1.5 Miniemulsion polymerisation

Conjugated polymer nanoparticles dispersions of poly(9,9-dioctylfluorene) (**PFO**) and poly(9,9-dioctylfluorene-*alt*-bithiophene) (**PF8T2**) were prepared using various molar ratios (2, 5, 7.5, 10, 15, and 20 mol%) of 9,9-di(undecanesulfate)-2,7-dibromofluorene (**M3**) using the procedure described below and shown in **Scheme 2**, **Table S1** and **S2**. 9,9-Di(undecanesulfate)-2,7-dibromofluorene (**M3**) was dissolved in 20 mL of deionized water in a Schlenk tube and the solution was degassed by bubbling with nitrogen gas for 2 hours. A dry toluene solution (0.8 mL) of 9,9-dioctylfluorene-2,7-bis(boronic acid pinacol) ester (**M5**, 58.6 mg, 9.12×10^{-2} mmol), 2,7-dibromo-9,9-dioctyl-9H-fluorene (**M4**) (for **PFO** CPNs) or 5,5'-dibromo-2,2'-bithiophene (**M6**) (for **PF8T2** CPNs) and hexadecane (78 µL) was degassed with nitrogen for 1 hr. A stock solution of tris (dibenzylideneacetone) dipalladium (0) [$Pd_2(dba)_3$)] (18.5 mg, 20.0 µmol) and tri(o-tolyl)phosphine [P(*o*-tol)$_3$] (23.5 mg, 80 µmol) in 1 mL of anhydrous degassed toluene was prepared and 200 µL of this solution was added to monomer mixture. This resulting monomer/catalyst mixture was added to the degassed aqueous phase in the Schlenk tube and the entire mixture was sonicated using an microtip ultrasonicator (Sonic Vibra-Cell VCX750, Cole-Parmer 750W, 22% power) for 8 minutes, while cooling the reaction mixture in an ice water bath. The base, NaOH (800 µL of 1M solution), was added to the reaction mixture and it was vigorously stirred at 70°C for 24 hours. The obtained aqueous dispersion of CPNs was bubbled with nitrogen for 4 hours at 40°C to remove the toluene solvent and was dialysed using Thermo Scientific Slide-A-Lyzer Dialysis Cassettes (2K MWCO, 3 mL) immersed in a deionized water reservoir for 72 hours. The deionized water was decanted and replenished every 12 hours.

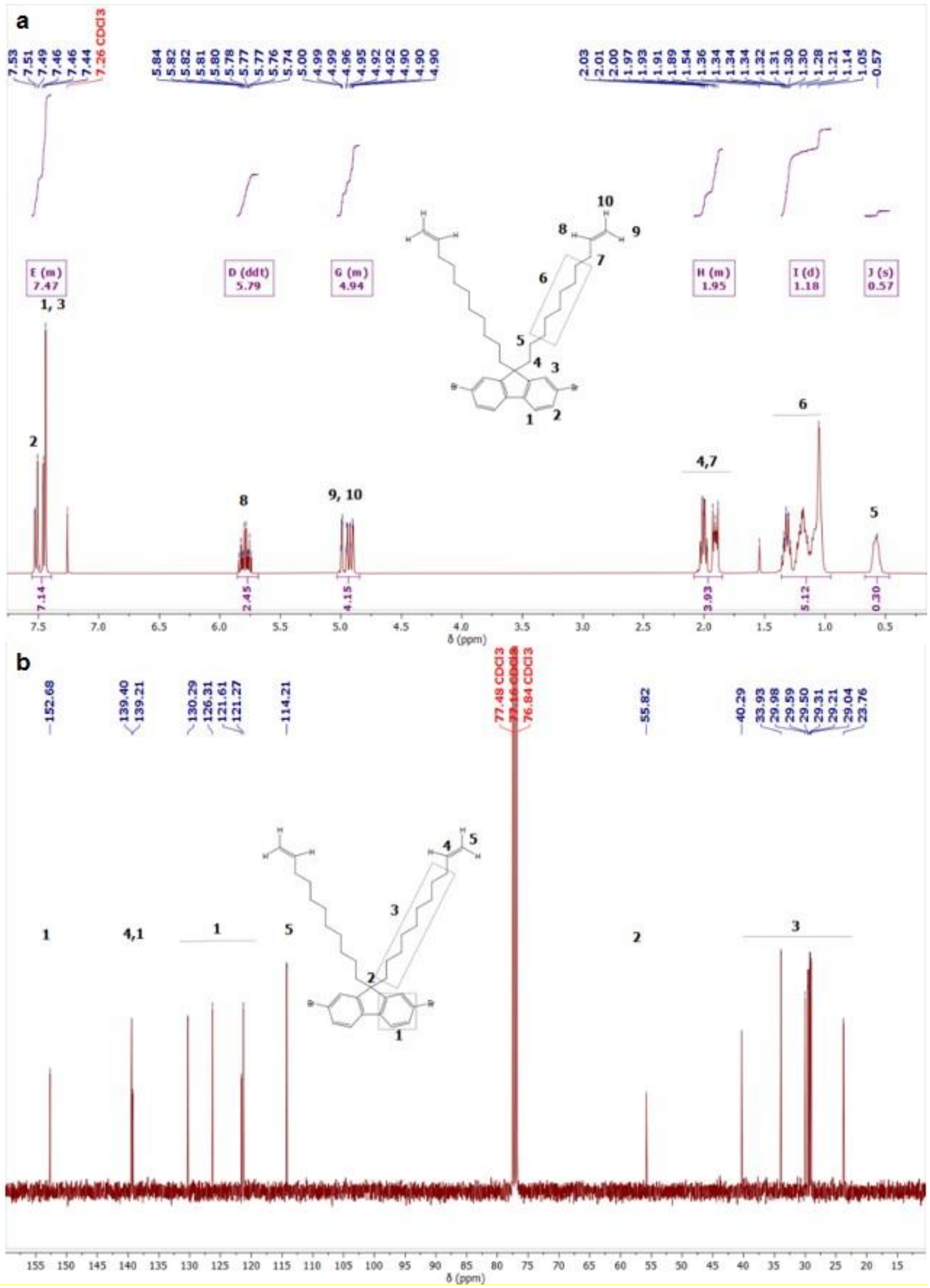

Figure S1. ^{1}H NMR spectra (**a**) and ^{13}C NMR spectra (**b**) of 9,9-di(undec-10-en-1-yl)-2,7-dibromofluorene (**M1**) in $CDCl_3$.

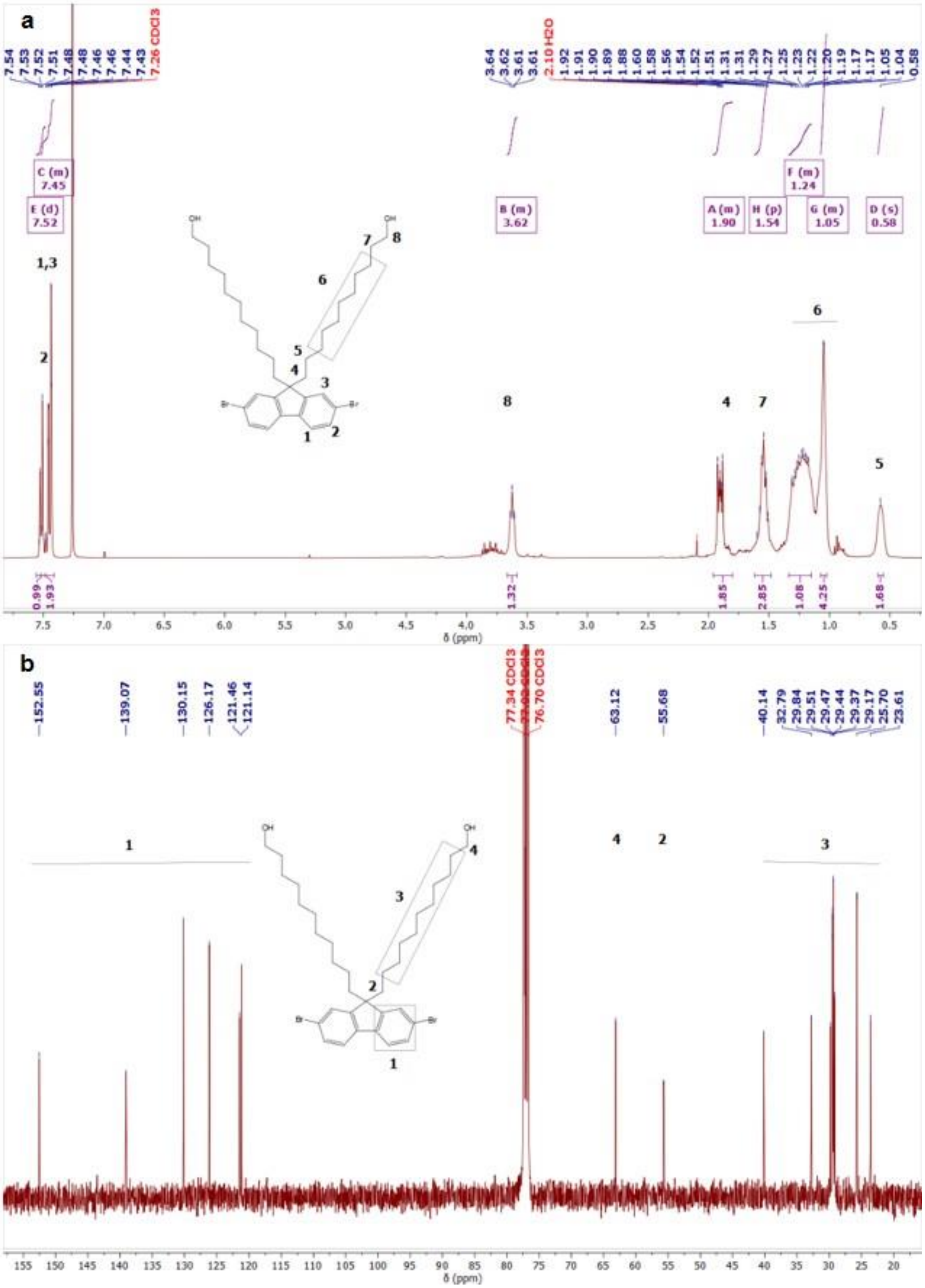

Figure S2. ^{1}H NMR spectra (**a**) and ^{13}C NMR spectra (**b**) of 9,9-Di(undecane-1-ol)-2,7-dibromofluorene (**M2**) in $CDCl_3$.

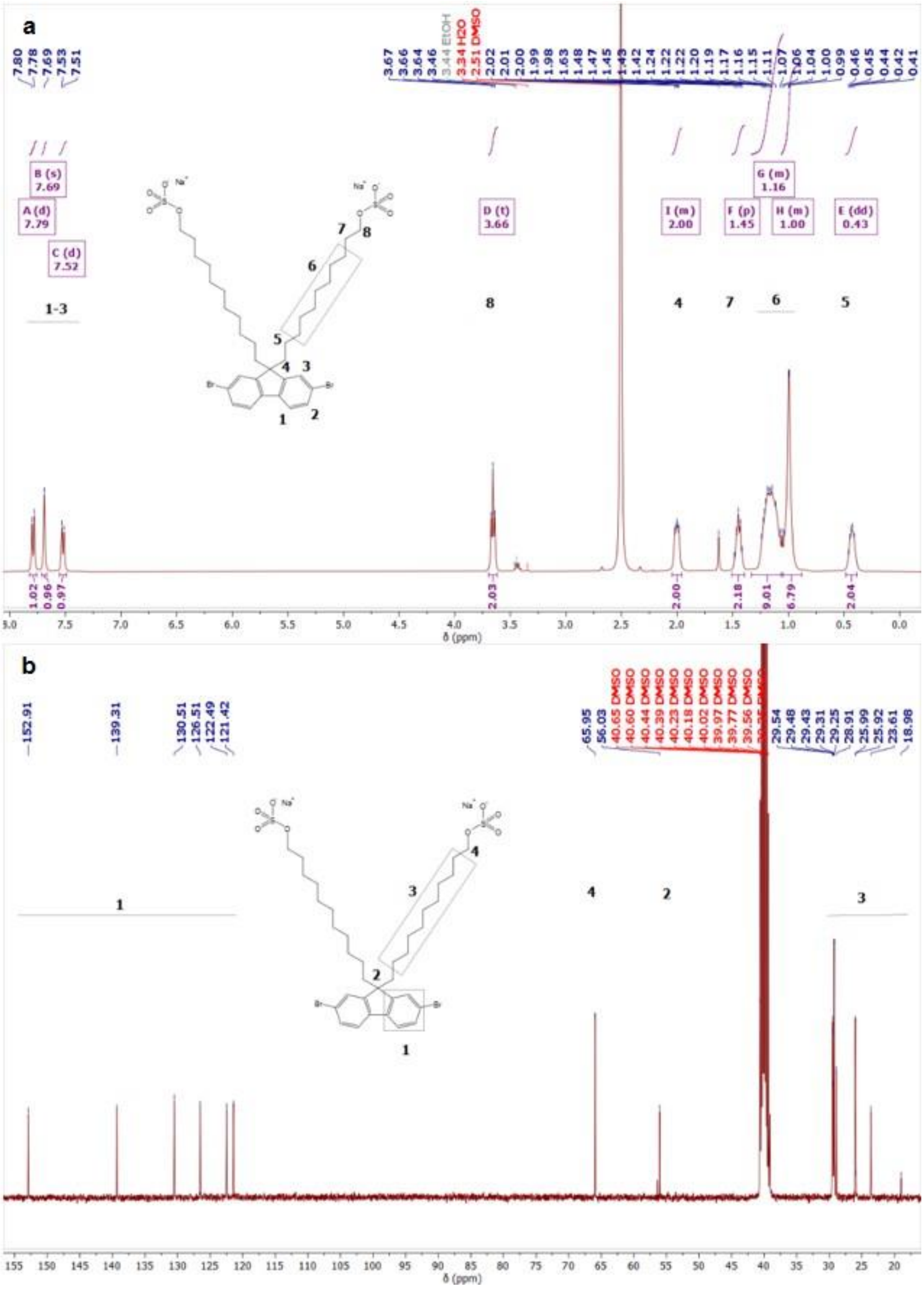

Figure S3. ^{1}H NMR spectra (**a**) and ^{13}C NMR spectra (**b**) of 9,9-Di(undecanesulfate)-2,7-dibromofluorene (**M3**) in DMSO-d^{6}.

S.2 Experimental method - Characterisation

S.2.1 Dynamic Light Scattering (DLS)

An aliquot (5 µL) of the aqueous dispersion of CPNs was dispersed in 1 mL of deionized water, vigorously mixed and injected into a quartz cell for analysis using a Malvern Zetasizer Nano ZS (633 nm red laser source). For determination of the surface potential, a specific zeta potential Dip Cell was utilised with two palladium electrodes immersed in 1 mL of the sample. All DLS measurements were conducted three times at 25°C and repeated after 1 month to examine the stability of the synthesised CPNs dispersions.

S.2.2 UV/Vis absorption and photoluminescence emission spectra

UV/Vis absorbance spectra (200-700nm) were recorded using a Varian Cary 5000 UV-Vis-NIR spectrophotometer by diluting 10 µL of CPN dispersions in 3.5 mL of deionized water. The dispersion was vigorously mixed and loaded into the quartz cell, which was placed into the UV/Vis spectrophotometer. Photoluminescence emission spectra were obtained using a Cary Eclipse Fluorescence Spectrophotometer (440 – 700 nm) by diluting 2 µL of CPN dispersion in 3.5 mL of deionized water.

S.2.3 Size Exclusion Chromatography (SEC)

The molecular weight of the polymers was analysed in THF (1 mL/min) by an Agilent 1260 Infinity equipment (2 x PLgel Mixed B, 3 µm 100 Å, 300 x 7.5 mm, PL1110-6320) fitted with a refractive index (Agilent 1260 Infinity Refractive Index Detector) and an ultraviolet detector (Agilent 1260 Infinity Variable Wavelength Detector, UV 254 nm). The systems was calibrated by low dispersity polystyrene standards (PL EasiCAL). To isolate the polymers from the dispersion 200 µL of CPNs dispersion was added to 1.2 mL of 1M solution of KCl in a 2 mL centrifuge Eppendorf tube. Subsequently, the sample was centrifuged at 14,000 rpm for 1 minute. Then, the supernatant was decanted, and the pellet of the precipitated polymer was dried in a stream of nitrogen for 1 hour. The polymer sample (*ca.* 1 mg) was dissolved in THF (suitable for HPLC, ≥99.9%) followed by filtering using a 0.45 µm syringe filter into a glass vial. The prepared sample was placed into the autosampler of the SEC instrument, and the measurements were carried at 35°C.

S.2.4 Attenuated Total Reflectance-Fourier-transform infrared spectroscopy (ATR-FTIR)

ATR-FTIR spectra were recorded in the range 600 – 4000 cm^{-1} with 256 scans using by Nicolet iS5 spectrometer by Thermo Fisher Scientific. After subtraction of the baseline, the spectra were normalised to a peak corresponding to C-H bending at 809 cm^{-1}.

S.2.5 Nuclear magnetic resonance (NMR) spectroscopy

Samples of the polymers were isolated as described in **S2.4** (10 mg for ^{1}H and 30 mg for ^{13}C NMR spectroscopy). These were dissolved in deuterated chloroform ($CDCl_3$, 1.0 mL) or deuterated dimethyl sulfoxide (DMSO-d^6, 1.0 mL) in a 1.5 mL Eppendorf tube. The samples were vigorously stirred using a vortex mixer until the polymer was completely dissolved, and it was filtered into a dried NMR tube. The prepared specimen was loaded into the NMR Bruker Advance Spectrometer 500 MHz and 100 MHz. For small molecules only 16 scans were required to record ^{1}H NMR spectra, but 64 scans were necessary to record the ^{13}C NMR spectra. The polymer samples of **PFO** and **PF8T2** were analysed using high-temperature ^{1}H NMR spectroscopy with 60 seconds of applied relaxation delay at 351 K. Abbreviations used to assign the ^{1}H NMR spectra are as follows: singlet (s), doublet (d), triplet (t), quartet (q) or multiplet (m).

S.2.6 Transmission electron microscopy (TEM)

TEM sample holders using graphene oxide deposited on a lacey carbon covered Cu grid (300 mesh, Generon) were cleaned using an air plasma from a glow-discharge system (2 min, 25 μA). These TEM grids were placed on to a droplet (10 μL) of a diluted solution of each of the CPN dispersions (at ~100 ppm solids) for 1 minute. After that, the excess solution was removed using blotting paper and the TEM grids were transferred by tweezers to the droplet of deionised water, and they were left to air-dry for 30 minutes. The TEM images were acquired in brightfield mode with an accelerating voltage of 120 kV using a Thermo Scientific, Talos L120C microscope. The average size of the CPNs were evaluated by the measurement of 100 particles for each sample using imageJ graphical image processing software.

Table S1. Summary of the applied molar ratios of functionalised sulfate monomer (**M3**), dibromide (**M4**), and aryl boronate pinacol ester (**M5**) used to prepare the **PFO** CPN dispersions.

Sulfate loading molar ratio (%)	Sulfate Fluorene monomer M3 (μmol)	2,7-dibromo-9,9-dioctyl-9H-fluorene M4 (μmol)	Boronic acid pinacol ester M5 (μmol)	Ratio of the 9,9-dioctyl-9H-fluorene units in PFO (x+y)	Ratio of the Sulfate Fluorene units in PFO (z)
**2.0**	3.7	87.5	91.2	0.980	0.020
**5.0**	9.1	82.1	91.2	0.950	0.050
**7.5**	13.7	77.5	91.2	0.925	0.075
**10.0**	18.2	73.0	91.2	0.900	0.100
**15.0**	27.4	63.8	91.2	0.850	0.150
**20.0**	36.5	54.7	91.2	0.800	0.200

Table S2. Summary of the applied molar ratios of functionalised sulfate monomer (**M3**), dibromide (**M6**), and aryl boronate pinacol ester (**M5**) used to prepare the **PF8T2** CPN dispersions.

Sulfate loading molar ratio (%)	Sulfate Fluorene monomer M3 (μmol)	5,5'-dibromo-2,2'-bithiophene M6 (μmol)	Boronic acid pinacol ester M5 (μmol)	Ratio of the 2,2'-bithiophene units in PF8T2 (x)	Ratio of the 9,9-dioctyl-9H-fluorene units in PF8T2 (y)	Ratio of the Sulfate Fluorene units in PF8T2 (z)
**2.0**	3.7	87.5	91.2	0.480	0.500	0.020
**5.0**	9.1	82.1	91.2	0.450	0.500	0.050
**7.5**	13.7	77.5	91.2	0.425	0.500	0.075
**10.0**	18.2	73.0	91.2	0.400	0.500	0.100
**15.0**	27.4	63.8	91.2	0.350	0.500	0.150
**20.0**	36.5	54.7	91.2	0.300	0.500	0.200

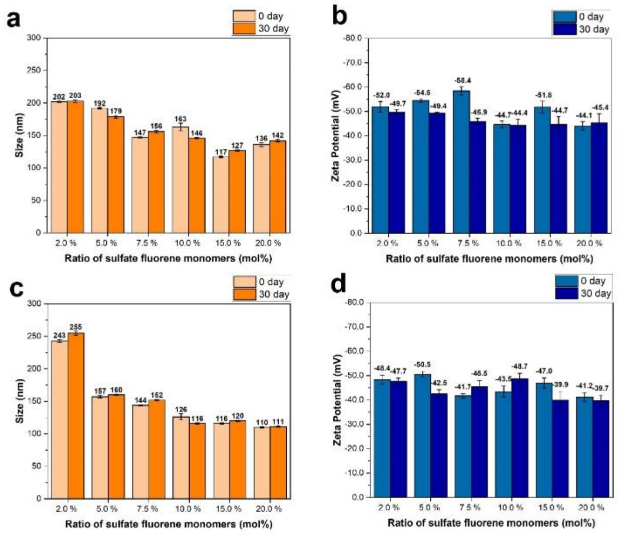

Figure S4. Comparison of the hydrodynamic diameter and surface potential (Zeta Potential) for **PFO** (**a** and **b**) and **PF8T2** (**c** and **d**) CPNs dispersed in water as synthesised and after storing for one month.

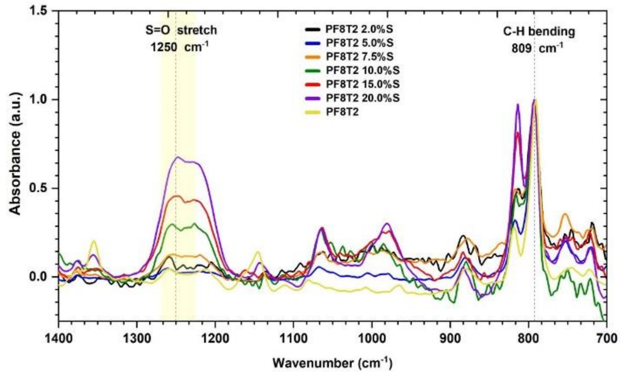

Figure S5. ATR-FTIR spectra recorded for **PF8T2** dispersions.

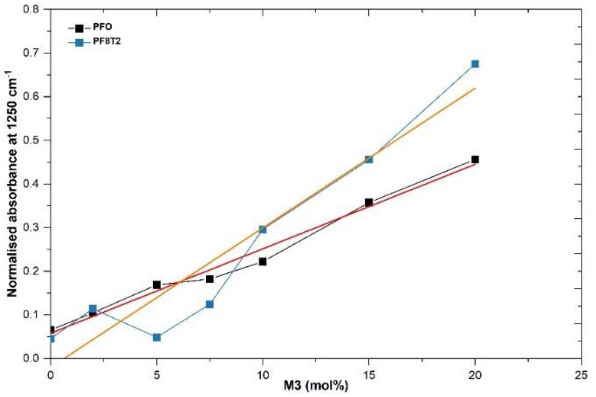

Figure S6. Relationship fitted by the linear regression between the intensity of the S=O stretch peak at 1,250 cm^{-1} for normalised ATR-FTIR spectra at 809 cm^{-1} for C-H bending.

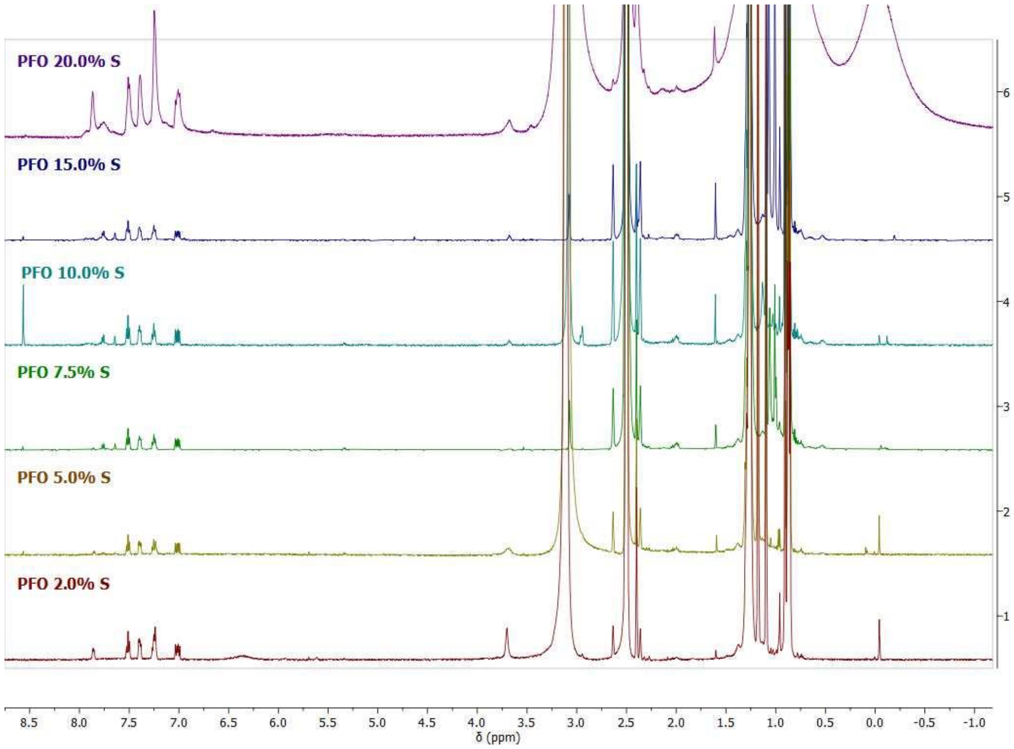

Figure S7. ^{1}H NMR spectra for **PFO** polymer with various ratio of the addition of the sulfate fluorene monomers (2.0 – 20.0 mol%) in DMSO-d^{6}.

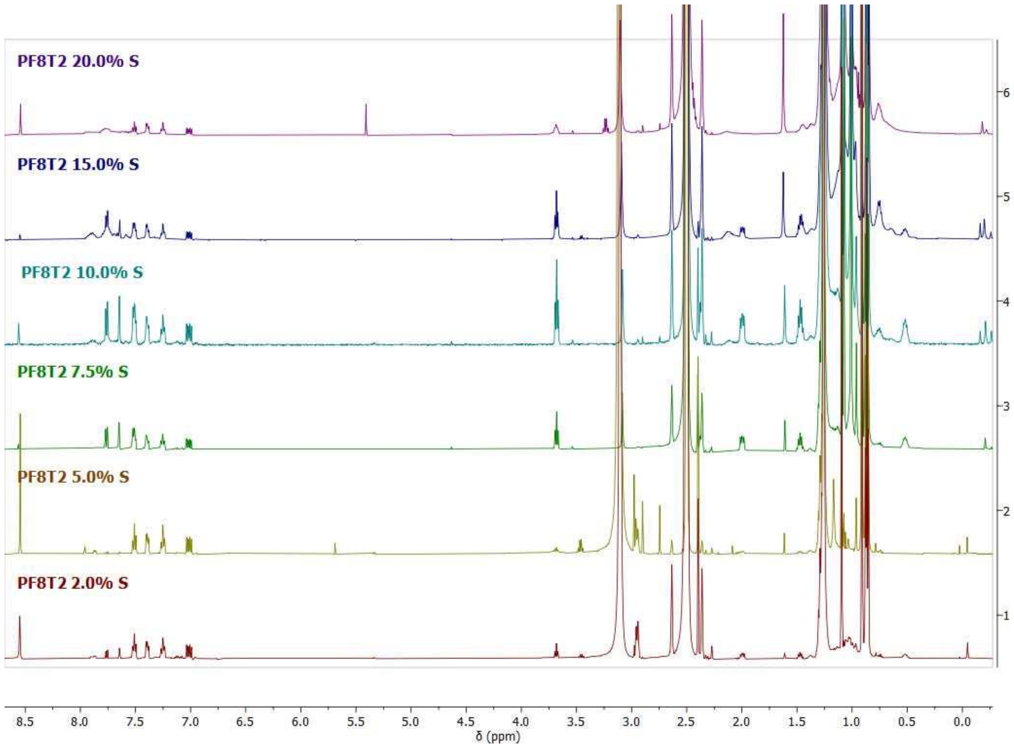

Figure S8. ^{1}H NMR spectra for **PF8T2** polymer with various ratio of the addition of the sulfate fluorene monomers (2.0 – 20.0 mol%) in DMSO-d^{6}.

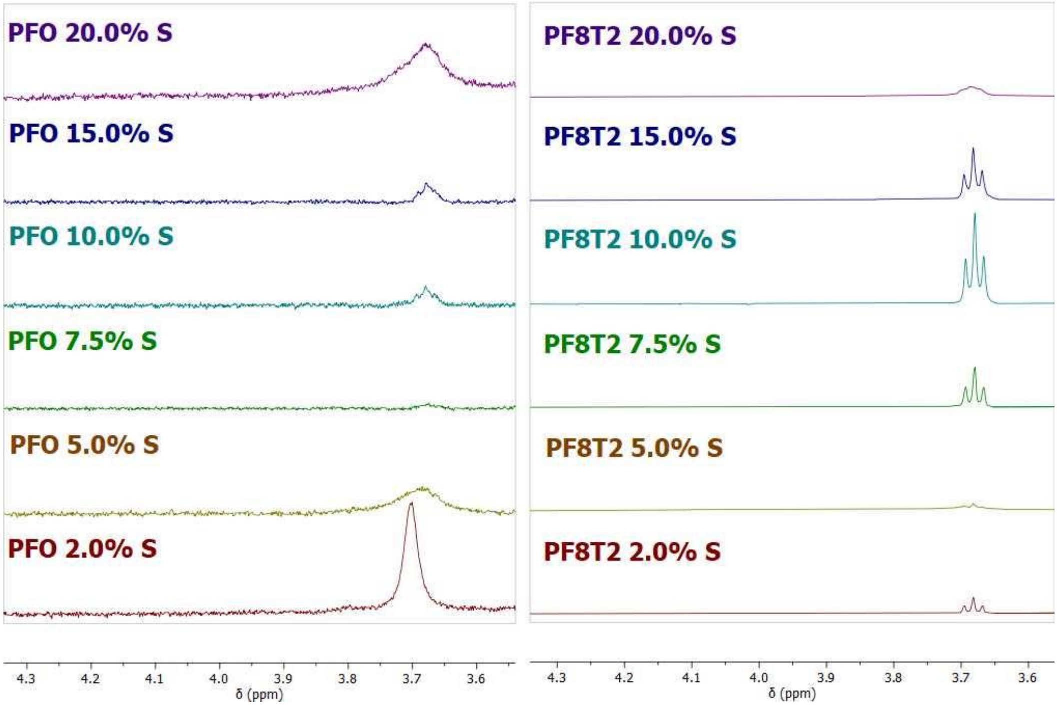

Figure S9. Comparison of stacked ^{1}H NMR spectra for **PFO** (left panel) and **PF8T2** (right panel) samples with various ratio of the addition of the sulfate fluorene monomers (2.0 – 20.0 mol%) with visible multiplet peak at δ 3.70 ppm assigned with the protons bond to the sulfate groups in in DMSO-d^6.

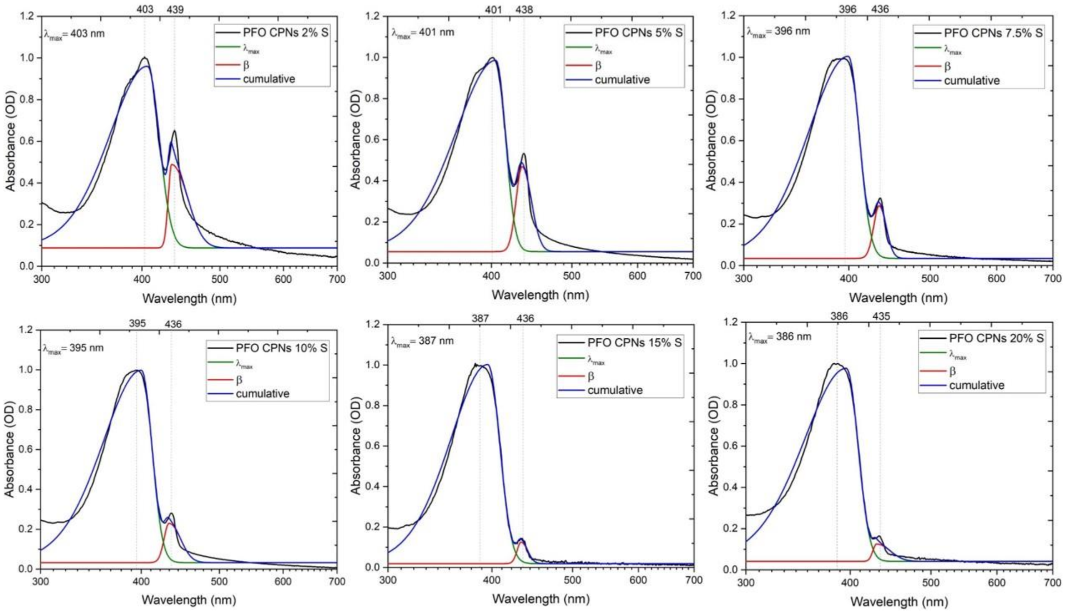

Figure S10. The deconvoluted absorption spectra for **PFO** CPNs samples with different sulfate fluorene units loading (2.0 – 20.0 mol%) fitted using a bi-gaussian function. Separated peaks for amorphous α-phase (λ_{max}) (green), ordered β-phase (red) and cumulative peaks of the bimodal function (blue).

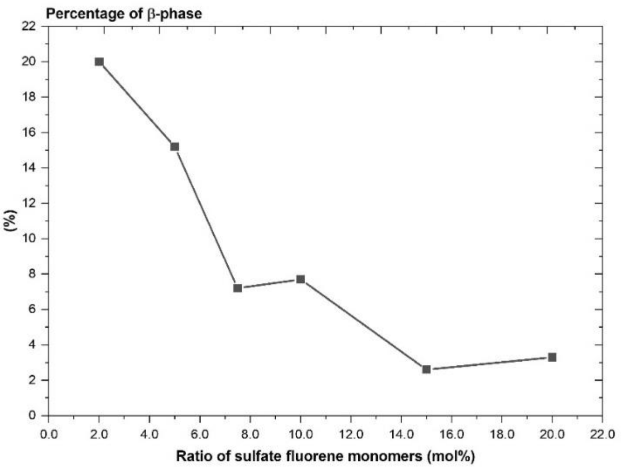

Figure S11. Proportion of β-phase for **PFO** polymers present in CPNs prepared with 2.0, 5.0, 10.0 and 20.0 mol% of sulfate fluorene monomer (**M3**).

Table S3. Summary of the integrated area of the absorption peaks for the amorphous α-phase (λ_{max}) and the ordered β-phase with the respective percentage of β-phase in the sample.

Sample code	Sulfate loading molar ratio (%)	Integration area of α-amorphous phase	Integration area of ordered β-phase	Percentage of β-phase
**PFO CPNs 2.0% S**	**2.0**	61.33	12.24	20.0%
**PFO CPNs 5.0 %S**	**5.0**	62.87	9.58	15.2%
**PFO CPNs 7.5 %S**	**7.5**	65.64	4.71	7.2%
**PFO CPNs 10.0%S**	**10.0**	65.47	5.06	7.7%
**PFO CPNs 15.0%S**	**15.0**	64.32	1.68	2.6%
**PFO CPNs 20.0%S**	**20.0**	66.16	2.21	3.3%

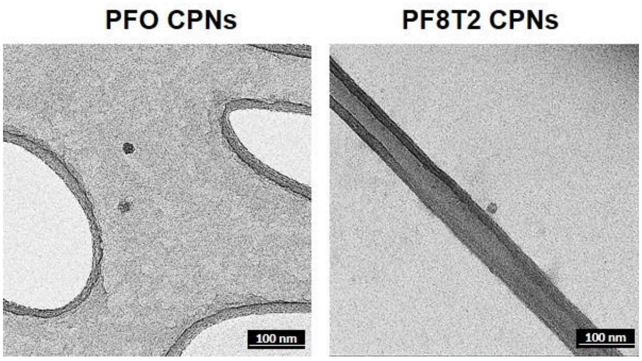

Figure S12. TEM images of the **PFO** and **PF8T2** with 20.0 mol% of the addition of the sulfate fluorene monomers acquired under 92 000x magnifications in bright field.

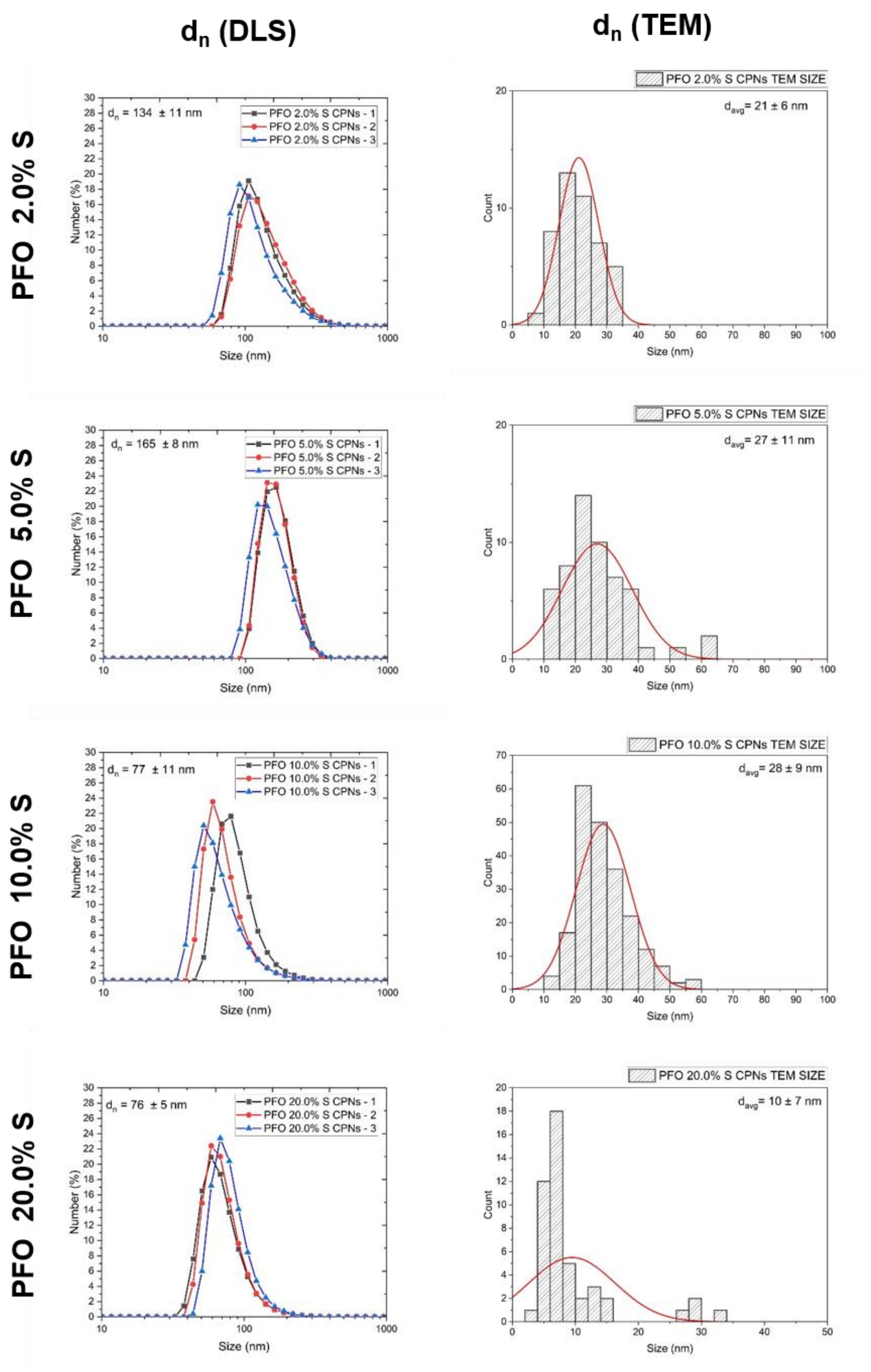

Figure S13. Comparison of the diameter of the **PFO** CPNs with addition of 2.0, 5.0, 10.0 and 20.0 mol% of **M3** measured by DLS (number distributions) and determined from TEM images (frequency histograms).

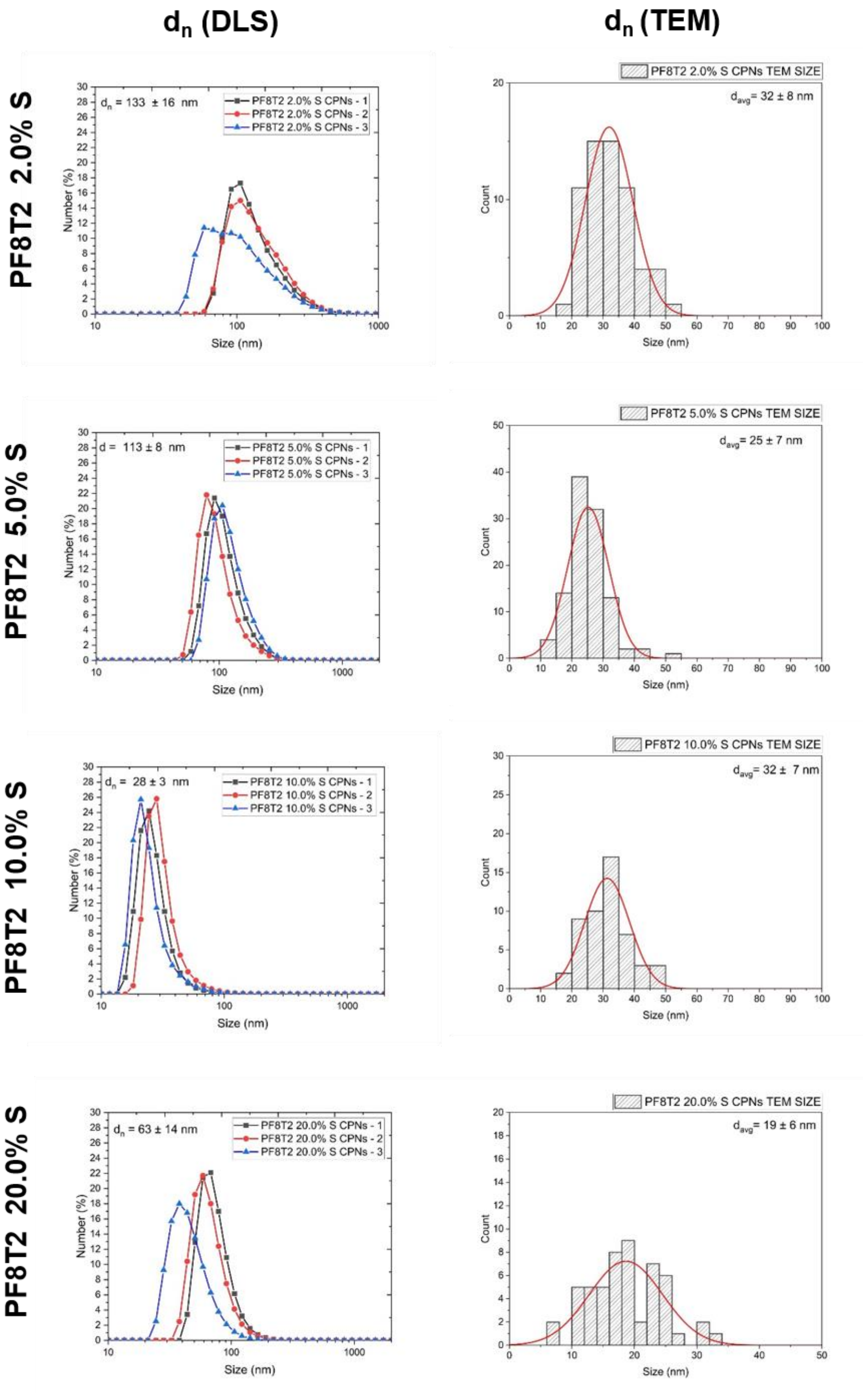

Figure S14. Comparison of the diameter for **PF8T2** CPNs with addition of 2.0, 5.0, 10.0 and 20.0 mol% of **M3** measured by DLS (number distributions) and determined from TEM images (frequency histograms).

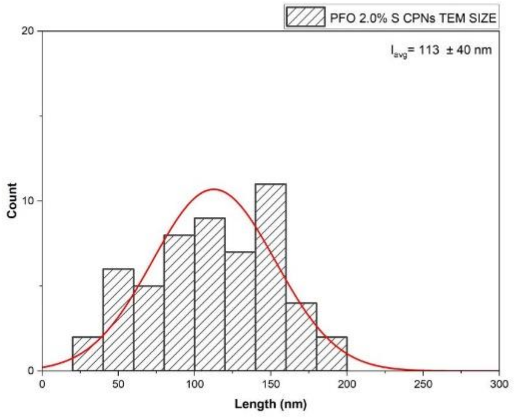

Figure S15. The frequency histogram distribution of the measured length of the rod-shaped nanoparticles observed in TEM for **PFO** CPNs prepared using 2.0 mol% of **M3**.
